# Sexual reproduction in land plants: an evolutionary perspective

**DOI:** 10.1007/s00497-025-00522-4

**Published:** 2025-05-12

**Authors:** Annette Becker, Xia Chen, Thomas Dresselhaus, Nora Gutsche, Stefanie J. Müller-Schüssele, Stefanie Sprunck, Günter Theißen, Sophie de Vries, Sabine Zachgo

**Affiliations:** 1 Institute of Botany, Justus Liebig University, Heinrich-Buff-Ring 38, 35392 Giessen, Germany; 2 Institute of Plant Sciences, Cell Biology and Plant Biochemistry, Universitätsstraße 31, 93053 Regensburg, Germany; 3 Division of Botany, Osnabrück University, Barbarastr. 11, 49076 Osnabrück, Germany; 4 Molecular Botany, Department of Biology, RPTU Kaiserslautern-Landau, 67663 Kaiserslautern, Germany; 5 Matthias Schleiden Institute/Genetics I, Friedrich Schiller University Jena, Philosophenweg 12, 07743 Jena, Germany; 6 Department of Applied Bioinformatics, Institute of Microbiology and Genetics, University of Göttingen, Goldschmidtstraße 1, 37077 Göttingen, Germany

**Keywords:** Sexual reproduction, Co-evolution, Sperm, RALF, HAP2, ROS, Ovule, Carpel, Double fertilization, MicroRNA evolution, Redox signaling

## Abstract

**Key message:**

We link key aspects of land plant reproductive evolution and detail how successive molecular changes leading to novel tissues and organs require co-evolution of communication systems between tissues.

**Abstract:**

The transition of water-dependent reproduction of algae to mechanisms with very limited water dependence in many land plant lineages allowed plants to colonize diverse terrestrial environments, leading to the vast variety of extant plant species. The emergence of modified cell types, novel tissues, and organs enabled this transition; their origin is associated with the co-evolution of novel or adapted molecular communication systems and gene regulatory networks. In the light of an increasing number of genome sequences in combination with the establishment of novel genetic model organisms from diverse green plant lineages, our knowledge and understanding about the origin and evolution of individual traits that arose in a concerted way increases steadily. For example, novel members of gene families in signaling pathways emerged for communication between gametes and gametophytes with additional tissues surrounding the gametes. Here, we provide a comprehensive overview on the origin and evolution of reproductive novelties such as pollen grains, immobile sperms, ovules and seeds, carpels, gamete/gametophytic communication systems, double fertilization, and the molecular mechanisms that have arisen anew or have been co-opted during evolution, including but not limited to the incorporation of phytohormones, reactive oxygen species and redox signaling as well as small RNAs in regulatory modules that contributed to the evolution of land plant sexual reproduction.

## Introduction

**Fig. 1:**
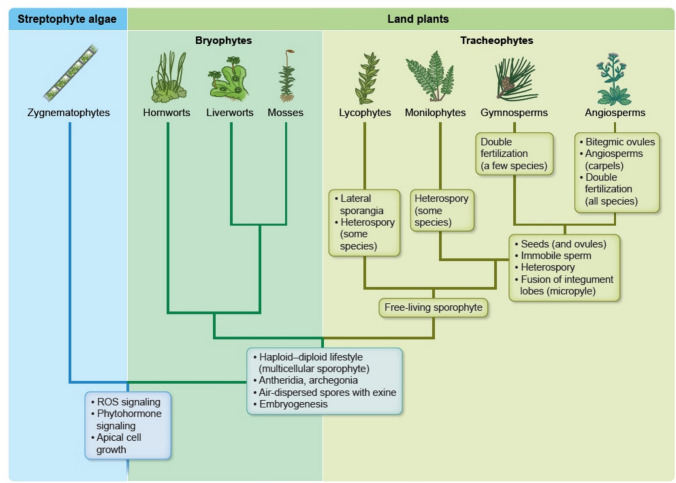
Schematic representation of the relationships of major land plant lineages and their Zygnemataophyte sister lineage. Major innovations in reproductive traits are indicated. Branch lengths do not indicate time scale, data for crown group origins time scale are from Harris et al. (2022)

One of the main challenges plants face on land as compared to life in water is their exposure to increased light intensity, water scarcity, and fluctuating environmental conditions. The most recent common ancestor of land plants and their sister lineage, the Zygnematophyceae algae (Fig. 1; Jiao et al. 2020; Hess et al. 2022), was likely a freshwater aquatic or hydro-terrestrial organism (Fürst-Jansen et al. 2020) that relied on liquid water for sexual reproduction, for example enabling its flagellate sperm cells to swim to the egg cells. The evolution of sexual reproductive organs in land plants has resulted in several traits that allowed an increasingly limited dependence on water during reproduction, which likely paved the way for long-term colonization of terrestrial habitats. This independence from water was achieved through a series of evolutionary novelties on different scales, including novel or re-wired signaling molecules, components of gene regulatory network, as well as novel tissues and organs required for sexual reproduction on land (Fig. 1). How the organ and tissue novelties connect with those on a molecular scale is still being actively investigated. Some connections found in recent years are highlighted in this review. While novel, adaptive traits arose in vegetative tissues, too; these were reviewed extensively elsewhere (Donoghue et al. 2021). Thus, in this review, we focus on land plant innovations with regard to reproductive tissues and organs as well as signaling pathways essential for reproduction.

The land plants (embryophytes) originated likely as a monophyletic lineage from within the grade of streptophyte algae, which include their algal sister lineage Zygnematophyceae (Fig. 1). Members of the Zygnematophyceae develop a single-celled zygote for dispersal or overwintering, and in several lineages a multicellular, haploid generation (Hess et al. 2022; Ohtaka and Sekimoto 2023; Permann and Holzinger 2024), which may have also been the most likely mode of alternation of generations in the common ancestor of land plants and Zygnematophyceae. This common ancestor had motile sperm cells and oogamy, and the multicellular gametophyte was capable of apical growth and branching (Bowman et al., 2022). Interestingly, there are exceptions to be found in the Zygnematophyceae where sexual reproduction involves the fusion of two non-flagellated gametes of similar size (isogamy), also referred to as conjugation. This suggests that, in addition to evolutionary novelties also reductive processes contributed to organismal diversification (Goldbecker and de Vries, 2024). All land plants feature traits not shared with the streptophyte algae that arose most likely in the stem group of all land plants after its divergence from the Zygnematophyceae algae. For example, land plants have a haploid-diploid lifestyle with a multicellular, diploid sporophyte, unlike in most streptophyte algae, whose only diploid cell is the zygote, that undergoes meiosis directly after fertilization without additional mitotic cell divisions. The spores of non-seed-bearing land plants then germinate into the gametophyte, which develops sexual reproductive organs (gametangia) of different sizes: antheridia, where typically smaller, motile sperm cells form, and archegonia where the typically larger, sessile egg cells develop. Flagella allow sperm cells to swim to the archegonia for egg cell fertilization, resulting in the formation of the zygote. This process requires, and in several species is often induced by rainwater, restricting the sexual reproduction of most non-seed-bearing land plants to the wet seasons that ensure a continuous layer of moisture.

The defining character of embryophytes (vascular plants, also known as tracheophytes, and bryophytes) is the formation of an embryo; a multicellular, diploid structure that is nutritionally and developmentally dependent upon continuous maternal tissues (Graham 1996), and in which the sporophyte body plan initiates (Ligrone et al. 2012). In bryophytes (hornworts, liverworts and mosses), the post-embryonic phase of development is limited, and the sporophyte remains attached to the gametophyte. The main bryophyte genetic model plants are *Physcomitrium patens* (moss)*, Marchantia polymorpha* (liverwort) and *Anthoceros agrestis* (hornwort). A major advantage of sexual reproduction including a multicellular sporophyte is that a significantly higher number of progeny can be produced in the sporangium as desiccation tolerant spores in comparison to an algal zygote. Already in 1908 it was suggested that the high number of offspring of a biphasic generation cycle may compensate for the transient water availability on land (Bower, 1890).

Tracheophytes include lycophytes (comprising lycopods or club mosses, Isoetales or quillworts and allies and Selaginellales or spike mosses), monilophytes (ferns, psilophytes and horsetails), and the seed plants (spermatophytes), composed of gymnosperms (including conifers, *Ginkgo biloba*, Gnetales and Cycadales) and angiosperms (Fig. 1). The arguably most important tracheophyte genetic model systems are *Ceratopteris richardii* (fern, an emerging model system), *Arabidopsis thaliana* (thale cress) and *Solanum lycopersicum* (tomato), both dicotyledonous angiosperms (flowering plants), and *Oryza sativa* (rice) and *Zea mays* (maize), both monocotyledonous angiosperms. In lycophytes and monilophytes, the sporophyte is dependent on the gametophyte early in its development, whereas female gametophytes in seed plants even develop within the sporophyte reproductive organs. Consequently, megaspores are no longer released but develop directly into gametophytes on the sporophyte. The sporophytic generation of tracheophytes is free-living, larger in size and morphologically more complex than the gametophytes, while the gametophytic generation is successively reduced, such that in around 70% of flowering plant species, the female gametophyte (embryo sac) consists of only eight cells (polygonum-type) and the male gametophyte (pollen grain and pollen tube, respectively) of three cells; one vegetative tube cell and two generative sperm cells. Sporangia of the Lycopodiaceae, like those of all lycophytes, develop laterally (relative to the stem) in the axils of specialized leaves termed sporophylls. In some members of the family, the sporophylls are similar to the vegetative leaves and co-occur with them on shoots that are indeterminate, i.e., with continuous growth. In other lycophyte family members, the sporophylls differ in size or shape from vegetative leaves and are aggregated into a terminal shoot system that is determinate, meaning that it terminates growth after formation. This determinate reproductive shoot, consisting of a terminal aggregate of sporophylls with associated sporangia, is known as a strobilus or cone.

Because of their exposed position on specialized leaves (sporophylls) and habitat expansion towards dryer regions, the reproductive structures of seed plants became more prone to dehydration than those of other vascular plants. One adaptation to alleviate the detrimental implication of dehydration was the emergence of ovules where integuments protectively surround the megasporangium, in which a megaspore will develop into the female gametophyte. Additionally, transition from flagella-bearing sperm cells (Renzaglia and Garbary, 2001) that require a moist environment to immobile sperm cells that are well protected within pollen grains and thus adapted to become airborne or take rides on pollinators occurred in conifers, gnetophytes and angiosperms. These steps played a pivotal role to render seed plant sexual reproduction fully independent of a moist environment. After fertilization, ovules will develop into seeds consisting of the embryo that arises from the fertilized egg cell, an ephemeral nutritive tissue that supports the growth of the embryo, and the protective layer of the seed coat, which is derived from the integument(s). This evolutionary novelty will be discussed further below in more detail. The trend to cover up the female gametophyte culminates in the carpel of angiosperms that protectively surrounds the ovules. The carpel confers a multitude of advantages to angiosperms and is thought to provide major contributions toward the dominance of angiosperms, that is, in terms of species number and total plant biomass in most terrestrial ecosystems. The carpel protects the ovules from biotic and abiotic stress and provides a mechanical and biochemical barrier preventing inbreeding as the male and female reproductive organs are often in close vicinity in angiosperms. Carpels develop stigmatic tissue at the apex, which captures a large number of pollen grains containing the male gametes. After pollination, compatible pollen grains germinate and form pollen tubes that grow through the maternal tissue and are guided precisely to the ovules by biochemical signals (Scutt et al. 2006; Becker et al., 2020). This enables the parallel fertilization of a large number of ovules and thus highly efficient reproduction. After fertilization, carpels develop into fruits (in some cases with the participation of other floral parts) protecting the seeds during their development and once matured, they provide a mechanism to distribute the seeds, often over large distances (Knapp and Litt 2013).

The origin of the carpel co-occurs with the emergence of double fertilization in angiosperms. In this process, not only the egg cells fuse with a sperm cell, but a second sperm cell fertilizes the central cell in the female gametophyte (Dresselhaus et al. 2016; Sprunck 2020; Zhong et al. 2025). The latter initiates the development of the endosperm, a tissue that nourishes the embryo in eudicots and provides resources to the seedling during germination in monocots. The endosperm is triploid in most angiosperms, with genetic contributions from both parents (Köhler et al. 2021). This additional fertilization process is thought to ensure targeted allocation of nutrients to the offspring and prevents the waste of maternal resources if fertilization did not take place. While double fertilization occurs in all angiosperms, many Gnetales also carry out double fertilization, but resulting mostly in supernumerary embryos, without providing embryo-nourishing tissue (Sharma et al. 2021). In the following, we will dedicate specific chapters to the origin of the carpel and the evolution of double fertilization.

More recently, it has been shown that reactive oxygen species (ROS) play a major role as signaling molecules and/or in the remodeling of the cell wall in many of the processes briefly described above, ensuring successful plant sexual reproduction: For example, ROS generation is essential for the degradation of the male gametophyte nourishing tissue, the tapetum with effects on pollen maturation. Embryo sac cell identity and polarity in angiosperms is also strongly dependent on ROS signaling (Martin et al. 2013). Pollen—stigmatic tissue interactions and thus male gametophyte functions require ROS as mediators, as do pollen tube growth and burst (Zhou and Dresselhaus 2023; Sankaranarayanan et al. 2020). An extra chapter is provided detailing the evolution of ROS signaling in plant development and reproduction. In addition, phytohormones also play an important role in regulating reproductive development. We therefore provide an overview of the current findings on their functions across the green lineage.

## Evolution of pollen and loss of sperm mobility

The evolution of pollen as a vehicle to protect sperm cells from drying out and to transport and disperse them over partly long distances occurred in the lineage leading to seed plants. This innovation represents a key adaptation that allowed seed plants to thrive in diverse and even very dry environments. In all extant angiosperms the germinated pollen forms a tip-growing pollen tube that transports a pair of nonmotile sperm cells as a passive cargo to the female gametes located deep in female reproductive organs (Zhang et al. 2017; Zhong et al. 2025). Gymnosperms display a higher diversity of pollen morphology, an ancient form of haustorial pollen tubes and produce either mobile or immobile sperm cells (Renzaglia and Garbary, 2001; Williams 2009; Breygina et al. 2021). In summary, the evolution of pollen has eliminated the need for liquid water for the transfer of sperm cells from male to female reproductive organs and has contributed significantly to the extensive biodiversity seen in modern seed plants of today.

**Fig. 2:**
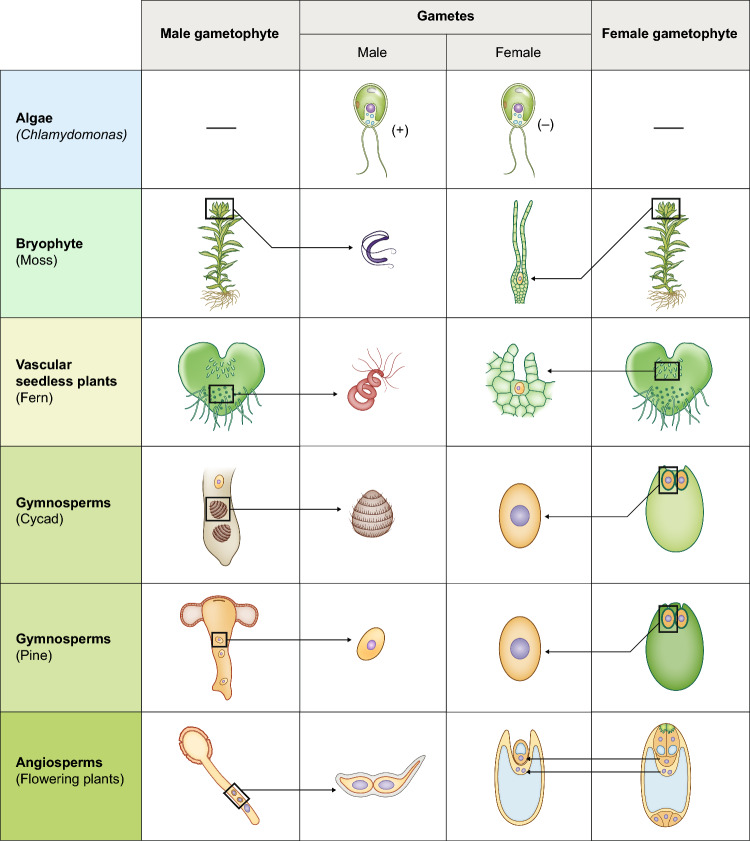
Comparison of examples for gametophyte and gamete morphology in *Chlamydomonas reinhardii* (with a haplontic life cycle with vegetative cells of two mating types) and major land plant lineages. In non-seed land plants, flagellated male gametes (sperm cells) are formed by antheridia, while archegonia contain egg cells (female gametes). In seed plants, gametophytes are highly reduced to male gametophytes (pollen) generating tubes containing sperm cells and archegonia with large egg cells in gymnosperms and embryo sacs containing two vacuolated female gametes (egg and central cell) in angiosperms (drawings not to scale)

**Fig. 3:**
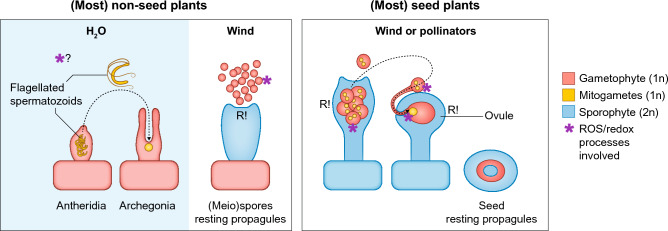
Simplified scheme showing reproductive modes of non-seed plants and seed plants. In non-seed plants mitogametes are produced in gametangia on the multicellular gametophyte plant body. Male gametes swim towards archegonia containing a single egg cell using two or more flagella. Involvement of ROS/redox processes is yet unclear. Sporophytes form a multitude of spores, which are desiccation-resistant (sporopollenin) and dispersed without the need for water. In seed plants, multicellular gametophytes are enclosed in sporophytic tissue (specialized sphorophylls; male = anthers, female = ovules) on the dominant sporophyte. Male gametophytes are reduced to 3 cells, the pollen, that is released from anthers and reach specialized structures on the carpel (angiosperms) or the ovule (gymnosperms) via the wind or pollinators. Without need of free water, the pollen grows a pollen tube, penetrating sporophytic tissues to reach the ovule bearing the reduced female gametophyte, the embryo sac, containing a single egg cell. Redox processes are involved in pollen formation, recognition, pollen tube growth, as well as ovule penetration and fertilization. The fertilized zygote forms an embryo inside the surrounding structures, which contribute to seed and fruit formation for effective dispersal and long-term survival

Green algae, such as the chlorophyte alga *Chlamydomonas reinhardtii,* rely entirely on water for their reproductive processes. Their isomorphic and mobile gametes (+ and − mating types; Fig. 2) typically contain two flagella supporting them to swim through water to achieve gamete fusion via agglutinins and the highly conserved fusogen HAP2/GCS1 (Pinello and Clark 2022; see below chapter on double fertilization). In algae, attraction of gametes is predominantly guided by gradients of chemical signals (chemo-attractants), often classified as pheromones (Frenkel et al. 2014). Bryophytes grow in mostly moist environments and show significant reproductive dependency on water. They, too, generate isomorphic spores forming male and female or hermaphroditic gametophytes (Fig. 3, McDaniel et al. 2013), that produce di-morphic gametes (Fig. 2). Small flagellated motile sperm cells develop in antheridia and strictly rely on external water films to swim towards the larger egg cells housed by archegonia. Bryophyte sperm cells are guided by chemotactic cues released by the archegonia. They detect the gradients of chemo-attractants and navigate towards higher concentrations directly towards and inside the archegonia, where fertilization occurs (Nath and Bansal 2015). In lycophytes and monilophytes, a gametophyte or prothallium (Fig. 2) produces both gametes (sperm and eggs) in specialized structures called antheridia (male) and archegonia (female), which are often located in proximity on the same gametophyte. Fertilization occurs when motile sperm guided by chemotactic cues swim through water or moist environment to reach the egg cell, leading to the formation of a new sporophyte (Boavida and McCormick, 2005).

The evolutionary transitions in the lineage that led to seed plants marks a significant step in the adaptation of plants to terrestrial environments. Crown group gymnosperms originated around 350 million years ago (MYA), of which several gymnosperm lineages became extinct. The extant gymnosperm lineages are morphologically very diverse, with four major remaining extant groups that contain the cycads and ginkgophytes, which are sister to the other gymnosperms, which include the conifers and gnetophytes (Yang et al. 2024). Gymnosperms are the first land plants exhibiting only heterospory (Fig. 3), by producing two distinct types of spores: microspores (male) and megaspores (female) in organs called male and female strobili (e.g. the cones of conifers), respectively (Linkies et al. 2010). This led to the emergence of separate male and female gametophytes. The female gametophyte of develops inside the megasporangium (nucellus) within the ovule providing additional protection and resources for the developing embryo after fertilization, while the male gametophyte was reduced to a pollen grain. Compared to non-seed plants, the gametophytes of gymnosperms are significantly scaled down and depend on the sporophyte for nutrition and protection.

As outlines above, the origin of pollen provided a crucial selective advantage for gymnosperms that encapsulate the male gametes (sperm cells) and facilitate their transport inside female reproductive organs (megasporophylls containing ovules) towards the two archegonia containing each one egg cell (Fig. 2). This innovation allowed gymnosperms to colonize drier environments and disperse their genetic material over greater distances via wind (Linkies et al. 2010). The protective outer layer of pollen grains, the exine, is composed of sporopollenin (see also next chapter), which makes them resistant to desiccation and mechanical damage, and ensures the viability of the enclosed sperm cells during transport.

After landing on the micropyle (small opening of the ovule) and adhesion at the megasporangial tissue of ovules, pollen grains of gymnosperms germinate and their tubes grow slowly inside the ovule. This ancestral pollen tube type is haustorial, in which the male gametophyte feeds itself from ovule tissues like a parasite. At this stage maturation of sperm cells may occur during months (Williams 2009). Most extant gymnosperm species generate—like all angiosperms—immobile sperm cells. Motile sperm cells still found in cycads and ginkgophytes (Fig. 2) are released from pollen tubes into a fertilization chamber, from where the sperm cells, containing up to a few thousand of flagella, swim towards the archegonia that contain the egg cell. This process is called zoidogamy (Poort et al., 1996). Conifers and gnetophytes generate also haustorial pollen tubes, but delivery of their immobile sperm cells to the archegonium containing the egg cell is entirely dependent on the pollen tube. This process is known as siphonogamy.

The presence of pollen and mobile sperm cells in cycads and ginkgophytes represents a blend of both ancestral aquatic reproductive traits and advanced adaptations to terrestrial life, displaying an intermediate stage in this evolutionary process. After both lineages separated from the remaining gymnosperms, all components of basal bodies and flagella were lost in sperm cells (Southworth and Cresti 1997). Moreover, the growth speed of pollen tubes increased enormously during further seed plant evolution. While pollen tubes grow very slowly in gymnosperms (< 20 µm/h), pollen tube growth rates range in the ANA-grade angiosperms (Amborella, Nuphar, and Austrobaileya) from approximately 80–600 µm/h to higher speeds in monocots and dicots with maize being world champion (10,000 µm/h). Accelerated pollen tube growth rate is considered as a critical innovation to strengthen competitiveness and appears correlated with the origin of callosic pollen tube walls and callose plugs, which are both missing in gymnosperms (Williams 2008; Williams and Reese 2019).

As described above, angiosperms completely lost sperm cell motility, rely entirely on pollen tubes for their transport and are released in immediate proximity of the female gametes (Dresselhaus and Franklin-Tong 2013; Zhong et al. 2025). They evolved an enormous complexity of pollen (surface) structure to optimize pollination efficiency, often leading to specific relationships with pollinators such as insects, birds, and mammals. The outer layer of the pollen grain is robust and drought-resistant, featuring complex patterns with a variety of shapes and sizes that are often species-specific (Katifori et al. 2010). This specialization aids in the recognition and attachment to pollinators, as well as protection from environmental stresses. Finally, it should be noted, that molecular studies have shown that pollen biogenesis and function in seed plants is associated with the co-evolution of numerous transcription factors and kinases essential for pollen and sperm development and their differentiation (Julca et al. 2021). Neo-functionalization is hereby caused by changes in gene expression pattern and DNA-binding capabilities enabling, for example, the algal ancestor of the MYB transcription factor DUO1 to recognize a new *cis-*regulatory element, which ultimately contributed to the evolution of sperm differentiation and the varied modes of sexual reproduction in the land plant lineage (Higo et al. 2018).

## The land plant innovation of sporopollenin

During transition to a terrestrial habitat, ancestral land plants adapted to novel, severe environmental challenges such as desiccation and harmful solar radiation. This adaptation is associated with the usage of sporopollenin, a synapomorphy of embryophytes, which mediates resistance of spores and pollen grains to novel stress factors, such as dehydration, UV-irradiation and mechanical stresses. This complex and intriguing biopolymer is extremely stable and can withstand mechanical, thermal, hydrostatic and biological stresses. Sporopollenin-like polymers are extremely stable, highlighted by the many fossil spores, dating back to approximately 450 million years (Grienenberger and Quilichini 2021) and precursors exist already in charophytes, protecting pre-meiotic zygotes (Permann et al. 2024). During land plant evolution, a shift in timing of sporopollenin deposition from zygotes to spores, explained by the sporopollenin-transfer hypothesis, resulted in the protection of these novel early land plant propagation units and later also of pollen grains from seed plants evolution (Graham, 1985; Grienenberger and Quilichini 2021).

In the microsporophylls and anthers of angiosperms, the tapetum supplies nutrients for developing microspores and also synthesizes the sporopollenin precursors for the protective exine of pollen grains. Many genes involved in the biosynthesis of sporopollenin have been identified mostly through genetic studies, but the detailed chemical structure of the polymer is poorly understood because of its inert nature (Quilichini et al. 2015). Recently, the first sporopollenin-structure of pine sporopollenin was uncovered. The main compounds contributing to pine sporopollenin are polyvinyl alcohol units, which are modified aliphatic-polyketide-derived and aliphatic C16 compounds (Li et al. 2019). Sporopollenin formation by polymerization and cross-linking to macromolecular components likely involves ROS (Rabbi et al., 2020). In Arabidopsis, PRX9 and PRX40, members of the land plant specific class III peroxidases, are essential for development of the exine, and homologs of PRX40 are found in vascular plants and *P. patens* (Jacobowitz et al. 2019).

## On the origin of the ovule and the seed

Seed plants currently dominate almost all terrestrial ecosystems in terms of total biomass and plant species richness. It is, therefore, reasonable to assume, that the hallmark of seed plants, the seed, represents an evolutionary novelty that significantly (but quite likely not exclusively) contributed to the evolutionary “success” of seed plants.

Important selective advantages that reproduction via seeds provides over that found in monilophytes, the extant sister group of seed plants (Fig. 1), is the independence from liquid water for fertilization and the capacity for embryo dormancy in changing environments (Linkies et al. 2010).

**Fig. 4:**
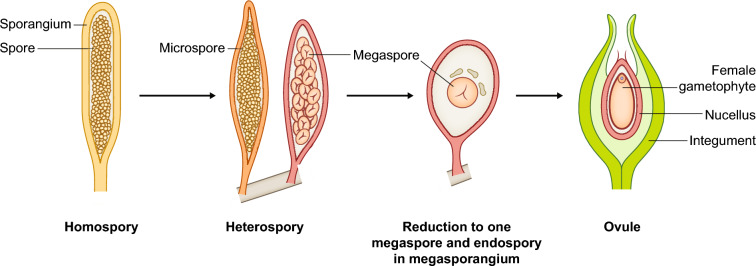
Likely steps in the origin of the ovule of seed plants. A highly simplified scenario is presented, starting from an ancestor with homospory, via heterospory, the reduction to one megaspore per megasporangium and endospory (retention of the megaspore), to the integumented megasporangium that is termed ovule

Seeds represent the endpoint of ovule development (Herr 1995), and understanding the origin of the seed consequently requires understanding ovule origin. Initially, ovules consist of a little stalk that bears the nucellus, that is the megasporangium of the seed plants. The nucellus (Fig. 2) is enveloped by one (in case of gymnosperms) or typically two (angiosperms) covering layers termed integuments (Linkies et al. 2010). From the perspective of developmental biology, an ovule hence might be interpreted as an unfertilized seed precursor; it is initially composed exclusively of diploid, sporophytic, maternal tissue. During development, a haploid megaspore is one of four cells generated within the nucellus by meiosis; the other three cells degenerate. The megaspore develops into a megagametophyte (female gametophyte). The mature megagametophyte of gymnosperms is multicellular; several archegonia typically develop within it, each producing one egg (Linkies et al. 2010). In most angiosperms, the mature female gametophyte, also called embryo sac, is seven-celled and eight-nucleate (Plygonum-type). In some early branching groups of angiosperms, however, the embryo sac is four-celled and four-nucleate (Nuphar/Schisandra-type), which is thought to be the ancestral type (Friedman and Ryerson 2009). One of the 7 (4) cells of the embryo sac represents the egg cell (Figs. 2 and 4).

The fertilized egg cell develops into an embryo. In angiosperms, a second sperm cell fertilizes a diploid central cell nucleus of the megagametophyte, giving rise to the development of a triploid endosperm.

The integuments of the fertilized ovule develop into the seed coat (testa) of the mature seed. A mature seed is well-suited for plant propagation even under adverse environmental conditions, as it is equipped with a drought-resistant embryo, an often nutrient-rich endosperm that nourishes it during early germination, and a protective seed coat. Sophisticated mechanisms regulating seed dormancy may favor germination only under favorable conditions (Linkies et al. 2010).

While the development of ovules and seeds is relatively well understood, the evolutionary origin of ovules and seeds is still largely unknown. We now know that during the origin of the angiosperm flower massive recruitment of gene regulatory networks (GRNs) controlling reproductive organ identity occurred, involving several orthologs of floral organ identity genes (Theißen and Rümpler, 2017). The situation is much different for ovules and the seed habit, because clear orthologs of ovule-identity genes have not yet been identified in non-seed plants (Gramzow et al. 2014; One Thousand Plant Transcriptomes Initiative, 2019). Initially, there may not have been an organ identity gene that could have been directly recruited for seed origin in the stem group of extant seed plants. The origin of the seed may thus have required more developmental genetic innovation than flower origin. In any case, the origin of the ovule and the seed is both one of the most significant events in the history of land plants and one of the remaining ‘black-boxes’ of plant evolution.

Even though extant seed plants are probably monophyletic (One Thousand Plant Transcriptomes Initiative, 2019), understanding the origin of ovules and seeds of the spermatophytes is hampered by the fact that seed-like structures may have originated several times independently in a probably paraphyletic group of ‘seed ferns’. The different groups of seed ferns (all extinct) probably originated from ‘progymnosperms’ (also all extinct), another paraphyletic group that produced gymnosperm-like wood but still had a fern-like mode of reproduction (Linkies et al. 2010). Ovules (or seeds, the difference is often not obvious) are quite well documented in the fossil record, beginning in the Middle to Late Devonian roughly 385–365 million years ago (MYA) (reviewed by Linkies et al. 2010). Unfortunately, however, it has not been possible to unequivocally clarify the origin and early evolution of the spermatophyte ovule so far, since the phylogenetic relationships of the different seed-producing taxa remain unresolved. Furthermore, parallel extinction of informative groups is obviously a major reason for our ignorance concerning ovule and seed origin.

Given its complexity, the spermatophyte ovule very likely originated in several steps (Fig. 4; Herr 1995; Bateman and DiMichele 1994; Linkies et al. 2010; Magnani 2018). The initial step probably involved the transition from homospory (production of just one kind of spores of equal size) to heterospory (production of two types of spores, small microspores and larger megaspores) in two morphologically divergent sporangia. The next step led to the retention of only a single megaspore in the megasporangium, probably to avoid competition for space and nutrients among four female gametophytes. Since each megaspore mother cell within a megasporangium produces four equal megaspores by meiosis, this requires the elimination of three of them. The single megaspore was not dispersed, but retained within the megasporangium, where it eventually develops into a megagametophyte containing an egg cell. Finally, integuments surrounding the megasporangium originated, possibly by fusion of some telomes (terminal branchlets of dichotomously branched axis). This way tightly locked, the megasporangium became fully indehiscent, and the ovule was established, providing a better protection for the egg in terrestrial environments and enabled the development of seeds.

Despite the plausibility of this scenario, it is still highly speculative, since intermediate states that could be unequivocally assigned to the lineage that led to extant seed plants are not known from the fossil record; moreover, the deep evolution of extinct spermatophytes is largely unresolved. Further, little is known about the molecular and developmental genetic changes that were involved in the origin of ovules.

Even though ovules are relatively complex structures, their origin may not have required massive genetic changes. Based on a detailed review of fossil evidence and Arabidopsis mutants, Herr (1995) recognized that mutations in single homeotic genes, such as *BELL1* (*BEL1*) in *A. thaliana*, encoding a homeodomain transcription factor, bring about some “primitive” (putative ancestral) features in ovules. For example, in *bel1-3* mutants, the inner integuments do not form (Herr 1995). It is therefore tempting to speculate that genes important for ovule structure may have been based on mutational changes in single homeotic genes. Magnani (2018) considered the loss of megasporangium dehiscence and hence retention of the megaspore, coupled to the partial degeneration of the nucellus to enable female gametophyte growth, as crucial processes during ovule origin. Whereas changes in B_sister_ MADS-box genes might have been important for nucellus degeneration, a different factor might have been involved in megasporangium dehiscence (Magnani 2018). Yet, another scenario is outlined by the “golden-trio hypothesis”. Based on studies on the fern *Adiantum capillus-veneris*, Bai et al. (2022) suggested, that the developmental program of the seed arose from a spatiotemporal integration of three physiological and genetic components: assimilate flow, stress responses mediated by the phytohormone abscisic acid (ABA), and stress-induced expression of the gene *LEAFY COTYLEDON1* (*LEC1*). Concerning the underlying physiological and genetic mechanisms of ovule origin, we are obviously quite far away from a consensus.

There is reason for optimism, however. In addition to an inconclusive fossil record and a great phylogenetic distance between extant seed plants and monilophytes, previous genetic hypotheses about ovule origin were hampered by the fact that high quality monilophyte genome sequences were lacking, and that genetic manipulation of respective species had not been established. This has changed now with the publication of whole genome sequences of several fern species (e.g. Li et al. 2018; Marchant et al. 2022; Fang et al. 2022) and protocols for their stable genetic transformation (e.g., Plackett et al. 2014).

## On the origin of the carpel

Carpels provide an extra tissue layer around the ovules and a specialized interface for pollen grain landing in combination with transmitting tissue that guides and supports pollen tube growth. The selective advantages of having a carpel may thus lie in the combination of (1) procuring an extra protective layer around the ovules, (2) serving as inbreeding barrier by providing the surface for the molecular selection processes allowing the discrimination of compatible from incompatible pollen grains and the fostering of germination and growth of the compatible ones, and (3) the further development of carpels into fruits after fertilization, enabling diverse and intricate mechanisms of seed protection and dispersal.

**Fig. 5:**
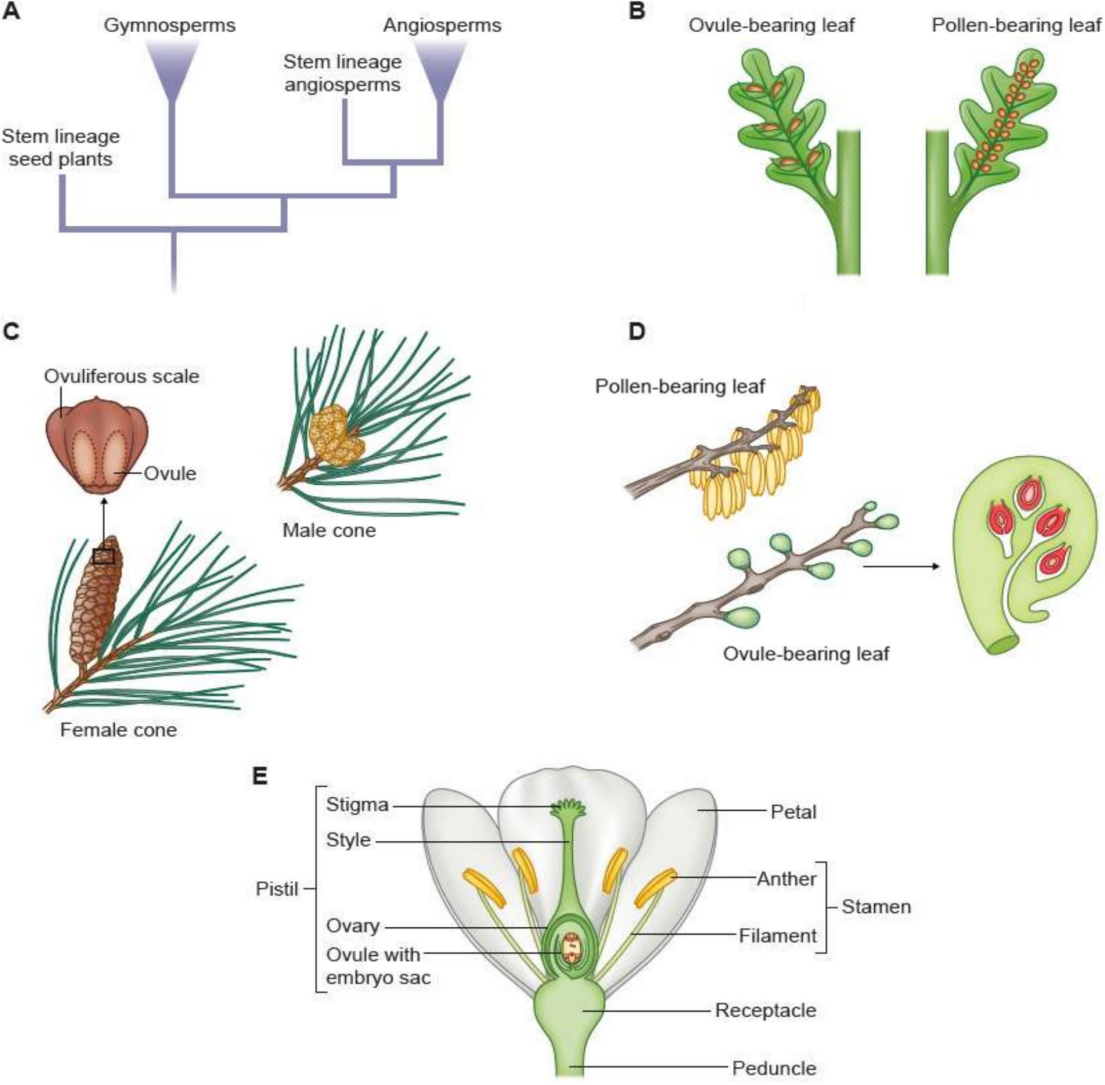
Morphological differences between of male and female gamete bearing sporophylls organs in extinct and extant seed plants. **A** Schematic phylogeny showing the relationships of extinct and extant seed plants. **B** Male and female sporophylls of Medullosa, a seed plant stem lineage relative, often referred to as seed fern. **C** Male and female sporophylls (cones or strobili) of the gymnosperm *Pinus ssp*. **D** Male (top) and female sporophylls of a Caytonia, a stem-lineage angiosperm relative. **E** angiosperm flower. Phylogeny redrawn after Scutt (2018), Medullosa drawn after Luthard et al., (2021), Caytonia redrawn from Frohlich and Chase (2007)

Gymnosperms and angiosperms diverged around 350 MYA (Stull et al. 2021), and extant angiosperms emerged as a monophyletic group around 130 MYA (Magallon et al., 2015). Within these 220 million years, several stem lineages angiosperms originated that already went extinct, such as the Mesozoic seed ferns of the Caytoniales (angiosperm stem-lineage relative, Fig. 5). In these seed ferns, leaves were bearing ovules and pollen on separate leaves, with pollination most likely mediated already by insects (Fig. 5, Dilcher 2010). Caytoniales, possibly the extinct sister group to angiosperms, enclosed their female reproductive organs in structures reminiscent to those in angiosperms, termed cupules, which are fleshy structures enclosing the ovules. These cupules have been viewed as carpel precursors previously, but are now more widely accepted as precursors of ovule integuments (Doyle et al., 2018). While the fossil record on carpel precursors is scarce and disputed (Bateman 2020), ancestral carpel traits can be inferred by combining morphological data from fossils and ANA-grade species of angiosperms, which include Amborellales, Nymphaeales and Austrobaileyales, that are sister to the remaining angiosperms, with phylogenetics. These studies propose that the ancestral carpel was cup or urn-shaped (ascidiate) with the carpel walls expanding upwards like a tube, as seen in the ANA-grade species (Doyle and Endress 2018). This is in contrast to what we see in many dicots, such as legumes, which have folded carpels. Further, few unfused carpels were united in the ancestral gynoecium (the collective of all carpels in a flower). Most likely, each of the carpels included a single, pendant ovule and was closed by mucilage, rather than by postgenital fusion, as observed for the majority of extant angiosperms. The style was lacking, and the stigma developed directly at the ovary apex, extending towards the base. This arrangement required the rather slow growing pollen tubes to travel only a short distance for fertilization, most likely without the need of specialized tissue like the pollen transmitting tract present e.g. in *A. thaliana* (Endress and Doyle 2009, 2015, 2011; Sauquet et al. 2017; Williams 2008). Like leaves, carpels (and gynoecia) develop along defined axis; style, stigma and ovary form along the apical-basal axis (Fig. 5E), the inner and outer surfaces are homologous to the adaxial and abaxial axes in the leaves, and the lateral expansion of carpel walls from central vascular bundles would be homologous to the medio-lateral axis in leaves. In *A. thaliana*, ovules form on the lateral side, at the adaxial surface from the placenta (Roeder and Yanofsky 2006).

Several hypotheses were proposed regarding the evolutionary origin of the carpel, but they mainly address how carpels could emerge in the middle of stamens and are thus concerned with reproductive organ arrangement and the origin of flowers. The “Out of Male/Out of Female” hypothesis suggests that a unisexual gymnosperm-like cone was converted to a hermaphroditic, flower-like structure by misexpression of floral homeotic genes. For example, a female cone with ovuliferous scales would show ectopic expression of floral homeotic B genes, which specify male reproductive organ identity, at its margins. Conversely, a male cone would lose B gene expression in its center. Both scenarios lead to a cone-like structure with stamen in basal and female organs in apical position, requiring only changes in the *cis*-regulatory region of homeotic genes. If this hypothetical cone was then flattened, it would resemble the inner (reproductive) whorls of flowers (Theissen and Becker, 2004). However, this hypothesis does not include assumptions on the origin of the carpel tissue. The “Mostly-Male” hypothesis suggests that hermaphroditic flowers originated from plants that produced male sporophylls with ectopic emergence of ovules. These ovules would have eventually been surrounded by male sporophyll-derived tissue, forming the carpel (Frohlich 2003). Already earlier, a theory developed by Zimmermann in 1930 suggested the carpel enclosing the ovules by planation of a telome-like branching system to form a flat organ that then curls inwards surrounding the ovules (Zimmermann 1959). However, how a dome-shaped meristem that generates sporangia on its flanks, or how ovuliferous scale-like organs were turned into a tissue that firstly generates the carpel wall and later ovules often from a de novo formed meristem, was not approached until recently. Interestingly, several examples, mainly from within the carnivorous plant lineages, show how intricate shifts in the expression of the HD-III ZIP family genes, required for establishing adaxial/abaxial polarity, may lead to cup-shaped leaves instead of laminar ones (Whitewoods et al. 2020). These may provide the first clues towards gene expression shifts allowing an urn-shaped carpel-like organ to develop at the center of the flower (Goncalves, 2021). However, how this emerges to separate stamens from ovules, and how this tissue then surrounds the ovules remains unclear.

The transcriptional regulation of carpel development has been analyzed in detail in Arabidopsis (reviewed comprehensively in Herrera-Ubaldo and de Folter 2022), but such an analysis is lacking in phylogenetically distant angiosperms. However, phylogenetic analyses of the most important carpel developmental regulators revealed that only few homologs of the Arabidopsis carpel regulators existed in the last common ancestor of land plants, among those the transcriptional co-regulators LEUNIG and SEUSS and the auxin biosynthesis regulators of the STYLISH/SHI/SRS family. Novel homologs of the many well-characterized carpel developmental regulators appeared in the last common ancestor of seed plants, angiosperms and core eudicots (Pfannebecker et al. 2017a, b). This suggests that the gene regulatory network required for the origin of carpels assembled stepwise along the lineage leading to flowering plants (Becker et al., 2020). Most likely, already existing transcriptional networks connected over time to allow for the emergence of the carpel. In this context, it is interesting to note that several core eudicot specific developmental regulators that originated by whole genome duplications (WGDs) from genes involved in carpel development, are necessary to specify the lignification pattern of the Arabidopsis fruit’s dehiscence zone. This highlights an important role of whole genome duplications, and retention of duplicate genes based on sub- and neofunctionalization, for the origin of novel gene regulatory networks (GRNs), that may have been also essential for the emergence of the evolutionary innovation of the carpel.

## Evolution of gametic/gametophytic communication systems

Associated with the emergence of pollen and pollen tubes that protect and transport sperm cells as well as the loss of sperm mobility in most gymnosperm and all angiosperm species, novel and highly specific communication systems had to be established. Until recently, it was thought that green algae like *C. reinhardtii* that generate isomorphic and mobile gametes lack pheromones for communication and attraction—and depend on random interaction and mating of compatible cells—while anisogamous species like *Chlamydomonas allensworthii* use small secondary metabolites like lurlenic acid as pheromones for attraction and guidance (chemotaxis; Frenkel et al. 2014). A peptidergenic signaling machinery using ciliary ectosomes and amidated small peptides was now discovered *in C. reinhardtii* generating chemoattractants for mating type minus gametes (Fig. 2) that repels plus gametes (Luxmi et al. 2019; Luxmi and King 2024). With the enormous diversification of gymnosperms and especially angiosperms, multiple and highly species-specific communication systems had to be established between male gametophyes (pollen/pollen tubes) and the various interacting female reproductive organs and tissues, respectively (Dresselhaus and Franklin-Tong 2013). Pollen of diverse species may land on receptive ovules or stigmata, respectively, thus self-pollen needs to be recognized, and pollen germination of the own species has to be promoted while preventing self-fertilization and thus inbreeding. Polymorphic-secreted peptides and small proteins, especially those belonging to various subclasses of small cysteine-rich proteins (CRPs) and their receptors play center stage in the complex regulation of these processes in angiosperms (Qu et al. 2015; Zhou and Dresselhaus 2019; Kim et al. 2021; Baillie et al. 2024; Xue et al. 2024; Zhong et al. 2025), while little is known about communication systems in gymnosperms.

**Fig. 6:**
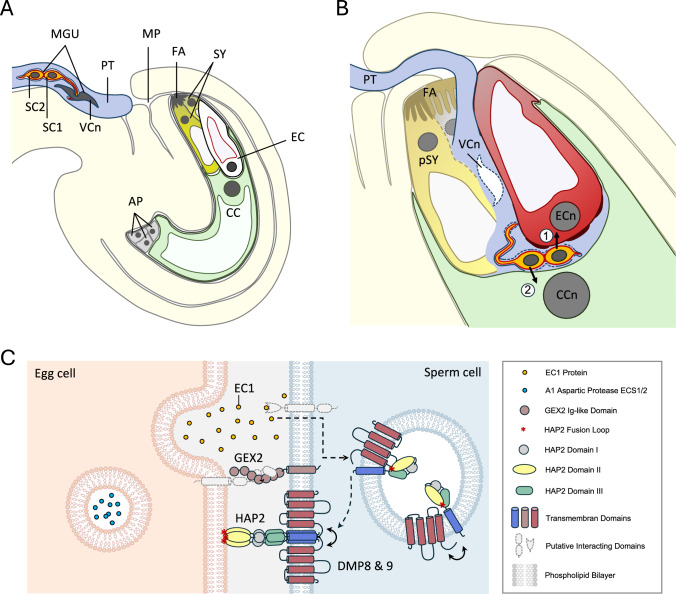
Double fertilization in the flowering plant *Arabidopsis thaliana*. **A** Arabidopsis ovule with approaching pollen tube. The pollen tube will grow through the micropyle that is formed by the inner and outer integuments of the ovule to deliver the two immotile sperm cells to the female gametophyte comprising the egg cell, two synergid cells, the central cell, and three antipodal cells. The two synergid cells (yellow) are located with their filiform apparatus close to the micropyle and flank the egg cell (red). The large central cell (green) is positioned in the center, and three antipodal cells (grey) are located at the opposing end of the female gametophyte. The two sperm cells are transported as part of a male germ unit: they are physically connected to each other by a common, transverse cell wall, and one sperm has a long, membranous projection that is wrapped around and partially embedded in the lobed nucleus of the vegetative cell. In addition, the peri-germ cell membrane, originating from the vegetative cell, surrounds the sperm pair (Sugi et al. 2024). **B** Ovule after pollen tube discharge. The two sperm cells are released and become trapped in a gap between the egg cell and the central cell. The peri-germ cell membrane has been stripped and one sperm cell attaches to and fuses with the egg cell (1), while the second sperm attaches to and fuses with the central cell (2). **C** Fertilization-essential proteins acting at the cell surfaces during gamete interaction and fusion. Abbreviations: AP, antipodal cells; CC, central cell; CCn, central cell nucleus; DMP, Domain of Unknown Function 679 Membrane Protein; EC, egg cell; EC1, Egg Cell 1; ECn, egg cell nucleus; FA, filiform apparatus; GEX2, GAMETE EXPRESSED 2; HAP2, HAPLESS 2; MP, micropyle; MGU, male germ unit; PT, pollen tube; pSY, persisting synergid; SC, sperm cell; SY, synergid cell; VCn, nucleus of vegetative cell

Initially, when pollen land on a compatible stigma in angiosperms, they absorb water and lipids from the stigma and hydrate, thereby activating metabolic pathways within pollen that trigger germination. Subsequently, pollen tubes grow via papilla cells through the style towards ovules, guided by attractants secreted from the ovule and egg apparatus (egg and synergid cells), respectively (Fig. 3). These processes are also summarized as the progamic phase. Tremendous progress has been made recently to understand the complex molecular interactions and cell–cell communication processes along the pollen tube journey (e.g. Cheung et al. 2022). The RAPID ALKALINIZATION FACTOR (RALF) family of peptides should be named as an example for secreted and specific CRPs. At least 50% of RALF family members play key roles in signaling events during the progamic phase via interaction with receptor kinases of the *Catharanthus roseus* receptor-like kinase 1-like (CrRLK1L) family (Zhu et al. 2021), LORELEI (LRE)-LIKE GPI-anchored proteins (LLGs) acting as co-receptors (Noble et al., 2022) and cell wall-localized leucine-rich repeat (LRR) extensin proteins (LRXs) (Mecchia et al. 2017; Moussu et al. 2020). While Chlorophytes do not possess RALF genes, liverworts, mosses and lycopyhtes contain 2–4 genes, this number is increased in ferns and gymnosperms and further increasing in angiosperms with, for example, 24 genes in maize and 37 genes in Arabidopsis (Campbell and Turner 2017; Abarca et al. 2021; Zhou et al. 2024). In Arabidopsis, at least four stigmatic sRALFs (RALF1/22/23/33) and seven pollen-derived pRALFs (RALF10/11/12/13/25/26/30) regulate species-specific penetration of compatible pollen tubes (Lan et al. 2023). RALF4/9 regulate signaling networks during pollen tube growth (Ge et al. 2017; Mecchia et al. 2017) and additional pollen tube-specific RALFs (RALF6/7/16/36/37) signal during both, exit of each one pollen tube from the transmitting tract and pollen tube rupture inside the receptive synergid cell (Zhong et al. 2022). Further specificity in pollen-pistil interaction is achieved, for example, by CRPs like LAT52, PCP B, PrsS in and S-locus cysteine-rich protein (SCR/SP11), which regulate pollen hydration and specificity of self-incompatibility (SI) mechanisms (Kim et al. 2021). These CRPs are all related to antimicrobial defensins and may have evolved from responses to pathogen invasion (Allen and Hiscock 2008; Dresselhaus and Márton 2009; Kessler et al. 2010). Notably, many eudicot plant families use co-evolved S-locus-specific S-RNase/S-locus F-box protein (SLF) modules for SI, which are also discussed to have originated from defense responses (Zhang et al. 2024). In addition to the evolution of highly specific communication systems for pollen tube germination, penetration and growth, their attraction is also regulated by polymorphic peptides or small proteins that are secreted from synergid cells of the female gametophyte (Fig. 6). So far, only few attractants have been identified. Eudicots appear to use polymorphic CRPs related to defensins, while grasses like maize evolved novel peptides not existing in other plants (for review: Higashiyama and Takeuchi 2015).

These examples are intended to provide an initial overview of the complexity of highly specific communication systems that evolved during the progamic phase in flowering plants. More molecular players and mechanisms have been discovered in recent years, especially in the model plant Arabidopsis (Zhong et al. 2025), while little is known in other angiosperms, and almost nothing is known in gymnosperms. Similarly, gametic/gametophytic communication systems remain to be elucidated in bryophytes and ferns. In conclusion, communication processes during the progamic phase in flowering plants—from pollen hydration towards sperm cell release—are highly specific and rapidly evolving. They appear to be at least partially originating from defense signaling mechanisms and are mainly regulated via polymorphic signaling peptides of several CRP families (Bircheneder and Dresselhaus 2016; Xue et al. 2024). Mutations in above-described peptide ligands, their receptor binding sites and downstream signaling processes likely represent a major driving force of speciation and reproductive isolation in flowering plants. This knowledge can now be used to overcome hybridization barriers and to generate novel crop plants (Lan et al. 2023). More research is required, especially in outside the seed plants to elucidate whether non-seed bearing land plants already use similar or different peptides for gametic/gametophytic communication and to which extent secondary metabolites, which can also be highly diverse, were replaced by proteinaceous molecules that play a role during the evolution of gametic/gametophytic communication systems in seed plants.

## The evolutionary innovation of double fertilization

In gymnosperms, the proliferating female gametophyte fulfills the function of an ephemeral nutrient tissue that supports the growth of the embryo (Linkies et al. 2010, Fig. 2). In contrast, the developing embryo in angiosperms is surrounded by the endosperm, a nutrient-rich tissue that results from a second fertilization process occurring in addition to sperm-egg fusion. This process, known as double fertilization, is a hallmark of angiosperm reproduction: while one of the two sperm cells released from a pollen tube into the female gametophyte fuses with the egg cell and forms the embryo, the second sperm cell fuses almost simultaneously with the central cell, from which the endosperm develops (Fig. 6, A and B; for review: Sprunck 2020). Of the two female reproductive cells that become fertilized, the egg cell is the female gamete sensu stricto: it is haploid, can unite with a sperm cell and passes on the genetic information of both parents to the next generation. However, the central cell is also a sexual cell and is referred to as the second female gamete, as it is also formed by meiosis and can unite with a sperm cell. Although endosperm development is an autonomously programmed process, independent of embryo development (Xiong et al. 2021), both fertilization products are necessary for development of a viable seed (for review: Lafon-Placette and Köhler 2014). Still, the origin and early evolution of the endosperm and the central cell as an additional sex cell in angiosperms has not yet been conclusively clarified (Friedman 1992; 1994; 2001; Baroux et al. 2002).

**Fig. 7:**
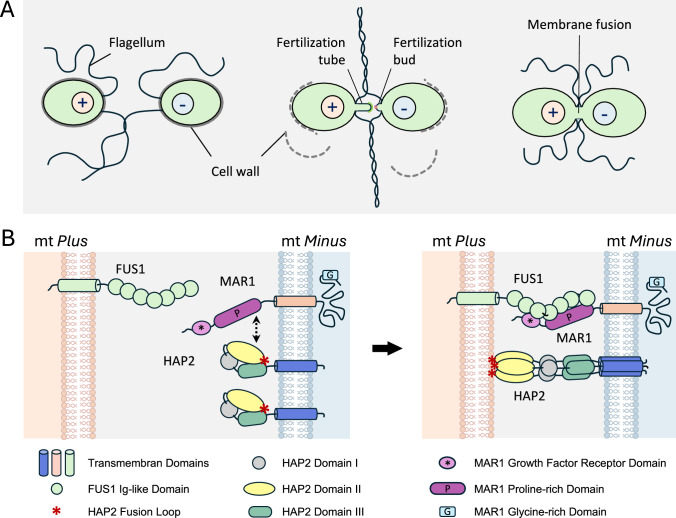
Sexual reproduction in the unicellular green alga *Chlamydomonas reinhardtii*. **A** Upon depletion of nitrogen and exposure to light, vegetative cells undergo a mitotic division to generate gametes that are either mating type *plus* or mating type *minus*. When the flagella of gametes of opposite mating types come into contact, mating type-specific agglutinins cause the flagella to adhere (left image). Flagellar adhesion triggers a signal transduction cascade that leads to the release of cell walls and the formation of mating structures (fertilization tube and fertilization bud), at the tips of which fertilization-relevant membrane proteins are located (center image). Gamete membrane adhesion and fusion occur between the tip of the mt(+) fertilization tubule and the apex of the activated mt(−) fertilization bud (right image). **B** Fertilization-essential proteins known to act on the plasma membrane of mating type *plus* and mating type *minus* gametes during gamete adhesion and fusion. Abbreviations: HAP2, HAPLESS 2; Ig-like, Immunglobulin-like; mt, mating type; MAR1, Minus Adhesion Receptor 1

While the ancestral mode of sexual reproduction in Viridiplantae is based on isogamy with identical-looking flagellated gametes (Fig. 7A), oogamy with a large, sessile egg cell that is fertilized by a smaller, motile sperm cell evolved in the chlorophytes in Volvox and within streptophyte algae in the Charophyceae (for review: Kirk 2006; Mori et al. 2015; Sharma et al. 2021). In land plants, true oogamy involving motile sperm is found in bryophytes, lycophytes, and monilophytes. Among the seed plants, the morphologically diverse gymnosperm orders also exhibit a wide range of reproductive strategies, including oogamy with unflagellated sperm cells or sperm nuclei, and the first occurrence of two parallel fertilization events. In *Ginkgo* and cycads, two flagellated sperm (spermatozoids) are released from the basal end of the haustorial pollen tube into the fertilization chamber and swim in the aqueous medium toward the archegonia. Examination of cultured female gametophytes of *Cycas revoluta* shortly before fertilization suggests that the female gametophyte is the source of this fluid, which can induce the release of sperm from the pollen tubes (Takaso et al. 2013). Only one sperm will penetrate the archegonial neck and fertilize the egg to form a zygote (Offer et al. 2023). A second fertilization event has been observed in Ephedraceae and Gnetaceae (Gnetales) (Friedman 1990; Carmichael and Friedman 1996; Friedman and Carmichael 1996). In *Ephedra*, the egg cell contains two nuclei, the egg nucleus and the ventral canal nucleus. Each of the two nuclei is fertilized by one of the two sperm nuclei contained in the generative cell of the pollen tube, which results in two fertilization products within the cytoplasm of the former egg cell (Friedman 1990). In *Gnetum gnemon*, the mature female gametophyte is coenocytic and lacks a differentiated egg cell. Here, each of the two sperm nuclei released from a pollen tube fuses with a separate, undifferentiated female nucleus within the coenocytic female gametophyte (Carmichael and Friedman 1996). Although both zygotes formed from the two fertilization events in *Ephedra* and *Gnetum* are viable and initiate embryo development, only one embryo will survive during the maturation of the seed.

The two fertilization events in the Gnetales are similar to the process of double fertilization in the angiosperms in that in both groups of seed-bearing plants the second karyogamy takes place between a second sperm nucleus from a single pollen tube and a sister nucleus of the egg nucleus (Friedman 1991). However, in contrast to the second karyogamy in *Ephedra* and *Gnetum*, fertilization of the central cell has evolved as a reproductive novelty in flowering plants (Friedmann 2001; Baroux et al. 2002; Butel and Köhler 2024).

Research on the two highly divergent model organisms *C. reinhardtii* and *A. thaliana* has contributed significantly to the identification of proteins that play important roles in gamete adhesion and fusion. In *Chlamydomonas*, initial adhesion between the flagella of mating type (mt) *plus* and *minus* gametes through mt-specific agglutinins initiates a signal transduction cascade that causes the release of the cell wall and the formation of tubular mating structures (Fig. 7A). Gamete membrane attachment and bilayer merger takes place at the tips of the mt(+) fertilization tube and the mt(−) fertilization bud, i.e. at sites of the plasma membrane where adhesion and fusion-relevant membrane proteins are localized (reviewed in: Snell 2022; Wilson 2008).

The comparison of the proteins or protein families involved in direct gamete interaction and fusion in *Arabidopsis* and *Chlamydomonas* shows that the molecular repertoire of these processes is based on evolutionarily conserved but also lineage-specific players (Figs. 6C and 7B). In both organisms, gamete membrane adhesion requires single-pass transmembrane proteins with a common ectodomain architecture constituted by seven Immunoglobulin (Ig)-like domains: the mt *plus*-specific FUS1 and sperm cell-specific GEX2 (Misamore et al. 2003; Mori et al. 2014; Pinello et al. 2021). Other proteins with FUS1/GEX2-like ectodomains are present in the clade of green plants, suggesting a conserved function of this protein family in the adhesion of compatible opposite-sex gametes (Pinello and Clark 2022). Membrane fusion is achieved by HAPLESS2 (HAP2), a broadly conserved Eukaryotic class II gamete fusion protein expressed in *Arabidopsis* sperm cells and in *minus* gametes of *Chlamydomonas*. HAP2 is a single-pass transmembrane protein that inserts in the outer leaflet of the opposing target membrane via an extracellular fusion loop, similar to viral class II fusion proteins that initiate fusion with host cells by inserting hydrophobic fusion loops into the host membrane (Fédry et al. 2017, 2018). The fact that HAP2 is conserved in multiple kingdoms besides algae and plants, including unicellular protozoa, cnidarians, hemichordates, and arthropods suggests that a HAP2-like fusogen was already present in the last common ancestor of all eukaryotes and represented a seminal innovation in the evolution of sexual reproduction (Wong and Johnson 2010; Fédry et al., 2018).

However, lineage- or species-specific mechanisms and proteins appear to be involved in regulating the activation of HAP2, or its subcellular localization: In *Chlamydomonas*, the species-specific Minus Adhesion Receptor 1 (MAR1) on *minus* gametes interacts with FUS1 on *plus* gametes but is also associated with HAP2 (Fig. 7B). FUS1-MAR1 receptor pair recognition initiates the fusion-promoting trimer formation of HAP2, suggesting a mechanism to ensure that local fusion of lipid bilayers is only triggered upon successful gamete membrane attachment (Zhang et al. 2021; Pinello et al. 2021). In contrast, in *Arabidopsis* sperm cells, HAP2 localizes mainly to cytoplasmic endomembrane compartments and requires regulated transport to the plasma membrane to render the sperm competent for fusion (Fig. 6C). Flowering plant-specific EGG CELL 1 (EC1) proteins, small cysteine-rich proteins secreted by the egg cell upon sperm cell arrival, trigger this process (Sprunck et al. 2012; Cyprys et al. 2019; Wang et al. 2022). Evidence for their high functional conservation during flowering plant diversification is given by the fact that an EC1-like protein from *Amborella trichopoda* can fully rescue the fertilization defect in the *Arabidopsis 5xec1* mutant. The finding that EC1 proteins are involved in the preferential fertilization of the egg cell suggests that this protein family is an evolutionary achievement of flowering plants to promote sperm-egg fusion during double fertilization (Wang et al. 2024). In addition, two DUF679 membrane proteins from *Arabidopsis* (DMP8 & 9), small multipass transmembrane proteins with specific expression in sperm cells, redundantly support gamete fusion, with a greater impact on sperm-egg fusion than on sperm-central cell fusion (Cyprys et al. 2019; Takahashi et al. 2018). Notably, DMP8 & 9 directly interact with HAP2 and are required for its trafficking to the sperm plasma membrane in response to EC1 (Fig. 6C) (Wang et al. 2022). While the genomes of *C. reinhardtii* and *P. patens* each encode one *DMP*, the *DMP* family is greatly expanded in *M. polymorpha* and seed plants (Cyprys et al. 2019; Wang et al. 2022). Evidence for a conserved function of DMP9-like proteins as HAP2 partner proteins in seed plants was provided (Wang et al. 2022), but the potential importance of the single DMP in *C. reinhardtii* for gamete fusion remains to be investigated.

After successful plasmogamy between egg and sperm cell, the two A1 aspartic acid proteases EGG CELL-SECRETED 1 (ECS1) and ECS2 are released from the fertilized egg cell to proteolytically cleave defensin-like pollen tube attractants (LUREs) and possibly other proteins relevant for double fertilization (Yu et al. 2021, Fig. 6C). However, further research is needed to clarify whether ECS1/2-related A1 aspartic acid proteases are important for sexual reproduction in gymnosperms, early land plants and algae.

## Recruitment and evolution of small RNAs to plant sexual reproduction

**Fig. 8:**
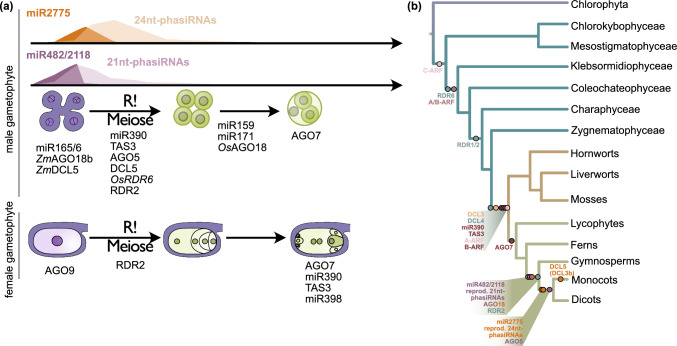
Small RNAs (sRNAs), non-coding *TAS* genes and the sRNA biogenesis machinery that play a role in sexual reproduction. On the top are those that contribute to male gametophyte development*,* and at the bottom those that contribute to female gametophyte development in plants. In monocots, expression miR482/2118 and resulting 21nt-phasiRNAs (purple) peaks during cell fate specification at 0.4 mm anther length in epidermal arc cells and anther cell layers, while miR2775 and the resulting 24nt-phasiRNAs (orange) peak during cell differentiation and the first meiotic stage, respectively (Zhai et al. 2015). Other sRNAs, *TAS* genes and sRNA biogenesis components involved are mentioned below the stages in which they play a role based on functional studies (after You et al., 2022). *Os* = *Oryza sativa*, *Zm* = *Zea mays*

Plant small RNAs (sRNAs) contribute to all aspects of plant life from stress responses to the control of plant reproduction. This is evident from studies concerning those proteins involved in their biogenesis. In brief (Zhan and Meyers 2023), generation of sRNAs requires Dicer-like proteins (DCLs) that act in a protein complex to process double-stranded RNA (dsRNA, linear or hairpin structures) into small molecules of 21–24 nt length. Some biosynthetic processes, e.g. the generation of trans-small interacting RNAs (tasiRNAs) and phased small interacting RNAs (phasiRNAs), also require RNA-dependent RNA polymerases (RDRs). Guiding sRNAs to their respective mRNA targets requires Argonaute (AGO) proteins. AGO, DCL and RDRs belong to large gene families (Belangér et al., 2023) and some of their members have a demonstrated role in the formation of reproductive structures (Fig. 8a). One example is the rice AGO5c protein MEL1; mutants of which show severe effects on the pollen mother cells and gametogenesis of the female gamete was disrupted both prior to meiosis as well as in the tetrad stage (Nonomura et al. 2007). Another example from *Arabidopsis* are mutants for *ago9,* which had multiple defects during female gametogenesis; among others, they exhibited abnormal gametic cells, of which only one underwent meiosis to become a haploid megaspore, megaspores that develop from differentiated cells, and ovules containing two female gametophytes, one 2-nuclear and one 1-nuclear (Olmedo-Monfil et al. 2010). The same study reported that *rdr2* and *dcl3* of *Arabidopsis* exhibited similar phenotypes.

In the following, we focus on sRNAs involved in the development of angiosperm reproductive structures and their evolution: microRNAs (miRNAs), tasiRNAs and phasiRNAs. miRNAs are primary sRNAs; they are encoded by *MIR* genes; their transcripts form long hairpin structures, primary miRNAs (pri-miRNAs) that a DCL1-containing complex cleaves to 20–24 nt miRNAs in a two-step process in the nucleus, in the cytoplasm miRNAs then associate with AGO proteins (Zhan and Meyers 2023). tasiRNAs and phasiRNAs are produced by binding of 21 nt or 22 nt miRNAs to non-coding or coding RNA transcripts in a one- or two-hit model (i.e. either one 22 nt miRNA targets the RNA target at one locus and sRNAs are generated downstream of the target site, or two miRNA target loci are available in the transcript and are targeted by 21 nt miRNAs and the transcript is processed in upstream direction from the 3’ target site); this will trigger conversion of a targeted RNA in dsRNAs via RDR6 and SGS3 (Fei et al. 2013; Liu et al. 2020). DCL4 will then further process the dsRNA into 21–24 nt long sRNAs in a phased manner (Fei et al. 2013; Liu et al. 2020). These phasiRNAs can later target the same (*cis-*targeting) or other transcripts (*trans*-targeting); the latter of which are called tasiRNAs.

One example that gained increasing attention over the last decade is the miRNA superfamily miR482/2118. First described as being involved in broad-spectrum immunity against bacterial, fungal and oomycete pathogens (Li et al. 2012; Shivaprasad et al. 2012; Ouyang et al. 2014; de Vries et al. 2015, 2018), its role in reproductive development was uncovered over the years. miR482/2118 is a sequence-wise highly variable miRNA family, which presence is conserved in angiosperms and gymnosperms, suggesting an origin in the last common ancestor of seed plants (de Vries et al. 2015; Xia et al. 2015). It likely originates from the pseudogenization of *Nucleotide-binding site-leucine-rich repeat* (*NBS-LRR*) resistance genes following an expansion event in the common ancestor of seed plants (Zhang et al. 2022). While miR482/2118 maintain targeting and silencing of *NBS-LRR* genes in dicots in overexpression and wildtype plants in vegetative tissues (Shivaprasad et al. 2012; de Vries et al. 2018), monocots express them in the epidermal arc of pollen, pointing to a function in pollen development (Zhai et al. 2015). miR482/2118 leads to generation of phasiRNAs in monocots (Shivaprasad et al. 2012; Xia et al. 2015) and there, 21nt phasiRNAs derived from miR482/2118 targeting are required for pollen development (Fig. 8a, Zhang et al. 2022). Particularly, in the first maturation stages of pollen, miR482/2118 accumulates in maize pollen in epidermal arc cells, this is followed by a sharp drop in abundance after the 0.4 mm stage coinciding with a peak of 21nt phasiRNAs in the anther cell layers (Zhai et al. 2015). While the exact function of these phasiRNAs is as yet to be determined, male sterility lines exhibit aberrant phasiRNA production (Zhai et al. 2015), and miR2118 deletion mutants show male and female sterility (Araki et al. 2020). Moreover, mutants of *Os*DCL4, associated with miR2118-derived phasiRNAs also showed a male sterility phenotype (Liu et al. 2007). In contrast to monocots, most tomato miR482/2118 family members were exclusively expressed in vegetative tissue, yet some members also showed expression during early and late inflorescence, anthesis and green fruits, too (Canto-Pastor et al. 2019). This suggests that some residual reproductive activity might be observed in dicots, although how widespread this is, remains unknown. An evolutionary scenario on how miR482/2118 became associated with male pollen maturation in monocots, could be that in the last common ancestor of seed plants both functions were present. This would agree with the high levels of miR482/2118-derived phasiRNAs present in male cones of *Picea abies* (Norway spruce, Xia et al. 2015). After the split of monocots and dicots subfunctionalization occurred, where in monocots miR482/2118 controls mainly reproductive development and in dicots the regulation of *NBS-LRR* genes became the main function.

In addition, monocot male gametophyte development requires miR2775 which triggers the production of 24 nt phasiRNAs (Fig. 8, Zhai et al. 2015). miR2775 expression is associated with monocot specific DCL5 (also known as DCL3b; Song et al. 2012, Fig. 8b) and peaks during the 1.0 mm stage, followed by 24 nt phasiRNA production between 1.5 and 2.5 mm stages (Zhai et al. 2015). Interestingly, DCL5 mutants confer male sterility in maize (Teng et al. 2020). While miR482/2118 and *NBS-LRR*-derived phasiRNAs are conserved in seed plants, 21 nt PHAS loci have been lost in dicots (Liu et al. 2020, Fig. 8b). In contrast, miR2775 appears to be angiosperm-specific, but DCL5 processing of 24 nt PHAS loci is specific to monocot (Xia et al. 2019; Liu et al. 2020; Fig. 8b).

Co-option and evolutionary changes in targeting are important aspects of miRNA-guided reproductive development. The miR390-TAS3 system is an interesting example: miR390 expression leads to the generation of tasiRNAs by binding and degrading the non-coding RNA *TAS3*. Their regulatory module is involved in various steps of *Arabidopsis* development, including reproductive development; miR390 is enriched in male meiocytes and it controls the correct formation of the megaspore mother cell during ovule primordium development (Su et al. 2017, 2020; Huang et al. 2020). In *P. patens,* miR390 suppresses development of buds and leafy gametophores, both required for sexual reproduction (Cho et al. 2012). While the miR390/TAS3 module, including the final target *B-ARFs*, was present in the last common ancestor of land plants, its two-hit model targeting emerged only after the split of bryophytes and tracheophytes and the required AGO7 first occurred in the last common ancestor of seed plants (Xia et al. 2017; Bélanger et al. 2023; Carrillo-Carrasco et al. 2023; Fig. 8b). Lack of comprehensive data from ferns and lycophytes makes it unclear when exactly the two-hit module evolved.

In *A. thaliana*, the transition from vegetative to reproductive tissue is dependent on miR156 targeting of *SPLs* (Wu et al., 2006; Gandikota et al. 2007). In tomato, not only the transition but also proper carpel development relies on the miR156/SPL module (Ferigolo et al. 2023). miR156 overexpression led, for example to suppression of ovary formation and production of ovules (Silva et al., 2014), and plants overexpressing both miR156 and GA20ox, showed phenotypes similar to 156OE lines, with supernumerary as well as partially fused carpels or ectopic aberrant pistil-like structures (Ferigolo et al. 2023). miR156 and miR529 are described as belonging to the same superfamily. miR529 have, however, mainly been reported from non-angiosperm lineages (with exception of some monocots), while miR156 appears across the green lineages (Cuperus et al. 2011). Functional data from the liverwort *M. polymorpha* highlights the importance of miR529 targeting of *SPL* genes for the initiation of gametangia development (Tsuzuki et al. 2019). On the contrary, miR156 is not expressed in *M. polymorpha* (Tsuzuki et al. 2016). In *P. patens*, on the other hand, miR156 regulates the miR390-*TAS3* module affecting bud formation and thus has an indirect effect on reproductive development (described above; Cho et al. 2012). Thus, while an *SPL*-miRNA targeting network exists across land plants and contributes to reproduction, it remains unclear whether non-flowering plants use the miR156/529-*SPL* targeting for control of the transition between gametophyte and sporophyte only or whether other reproductive phenotypes are also associated with it.

## Evolution of ROS–dependent mechanisms in development and fertility

ROS have often been considered as disadvantageous molecules inducing damage to cells. However, ROS fulfill useful biological functions (Halliwell 2006) in different contexts. This includes a crucial role of ROS in response to diverse stresses, but also functions during development and fertility (Mhamdi and van Breusegem 2018; Wacszczak et al., 2018; Smirnoff and Arnaud 2019; Sankaranarayanan et al. 2020; Mittler et al. 2022; Zhou and Dresselhaus 2023; Ali and Muday 2024). Fascinatingly, reproductive functions relate to both male and female gametophytes as well as sporophytic tissues. Thus, ROS-dependent mechanisms had to co-evolve with reproductive modes in land plants (Figs. 2, 3). In addition, these different reproductive functions are associated with several ROS types and subcellular compartments. This paragraph provides a synthesis of ROS in land plant reproduction from an evolutionary and redox biology perspective. Important biological functions of ROS are related to their roles as electron acceptor, enzymatic substrate, signaling molecule or their toxicity to pathogens (Halliwell 2006; Smirnoff and Arnaud 2019; Waszczak et al. 2018; Castro et al. 2021; Mittler et al. 2022). Here, ROS localization as well as temporal ROS level dynamics are important factors determining ROS functions.

Local ROS generation and scavenging rates are variable and responsive to specific scenarios. Inside cells, conversion of generated superoxide (O_2_^−^) via H_2_O_2_ to water proceeds fast via several enzymatic reactions (Mhamdi and van Breusegem 2018; Waszczak et al. 2018; Smirnoff and Arnaud 2019). Protein thiols are protected via a highly reduced pool of glutathione in all subcellular compartments containing a glutathione reductase (Meyer et al. 2021; Schwarzländer et al. 2016). In contrast, O_2_^−^ in the extracellular space can be specifically enzymatically generated by transferring electrons from cytosolic NADPH to extracellular oxygen via NADPH oxidases (*respiratory burst oxidase homologs*, RBOH, in plants). In the acidic apoplast, dismutation of O_2_^−^ to H_2_O_2_ (and O_2_) can occur enzymatically or non-enzymatically. Extracellular H_2_O_2_ generation can affect intracellular redox-states as re-entry of H_2_O_2_ can be facilitated via peroxiporins (Meyer et al. 2021, Palez-Vico et al., 2024).

Fundamentally useful functions for different ROS types were likely already present in the last common ancestor of land plants, based on phylogenetic and experimental evidence. According to current knowledge, these basic functions rely on ROS as (1) enzymatic substrates in the apoplast: Extracellular ROS such as H_2_O_2_ can increase and influence cell wall polymer formation in conjunction with local peroxidase activity whereas hydroxyl radicals lead to cell wall softening (Tenhaken, 2014). (2) Intracellular H_2_O_2_ acts as terminal electron sink for metabolic regulation. For example, electrons are transferred via peroxiredoxins to H_2_O_2_ to oxidize thiol switches during metabolic regulation of carbon fixation in photosynthesis (Yoshida and Hisabori 2023). (3) (Apoplastic) H_2_O_2_ signaling and defense functions: Intercellular signaling in response to abiotic and biotic stresses occurs by H_2_O_2_ waves in conjunction with Ca^2+^ influx and electric potential changes (Miller et al. 2009; Martiniere et al., 2019; Mittler et al. 2022; Fichman et al. 2023; Koselski et al. 2023). In addition, chitin-triggered oxidative bursts are evolutionary conserved in land plants (Lehtonen et al. 2012; Chu et al. 2023). Interestingly, immunity and developmental responses might be evolutionary related by a common origin of cell-surface receptors (Ngou et al. 2024). Generating receptor-like kinase and *rboh* mutants in bryophytes has revealed that the Malectin-like receptor kinase modules involved in tip growth (Westermann et al. 2019) and the receptor-like cytoplasmic kinase (PBL family)/RBOH module in oxidative burst generation are evolutionary conserved between liverworts and flowering plants (Chu et al. 2023; Hashimoto et al. 2023).

As land plant evolution has re-shaped the alternation of generations as well as the body plans of sporophytes and gametophytes (Fig. 3, Harrison 2017), existing ROS-dependent mechanisms experienced co-, sub- and neo-functionalization. Novel cell types such as guard cells of stomata possess a sophisticated H_2_O_2_ signaling network for rapid regulation in response to environmental changes (Dietz and Vogelsang 2022). Locally and temporally restricted ROS generation in the apoplast contributes to root lignification in the SCHENGEN pathway (Fujita et al. 2020). Notably, the balance between different ROS types (O_2_^−^ and H_2_O_2_) may contribute to cell identity decisions in meristems (Zeng et al. 2017). These processes have in common that they are based on spatial or temporal ROS gradients. Thus, spatio-temporal modifications of RBOH activity during development have been investigated using mutants of different RBOH isoforms in *A. thaliana* (Mhamdi and van Breusegem 2018). This revealed that specific local generation of ROS downstream of RBOH activity also contributes to flowering plant reproduction at different levels and stages (Mhamdi and van Breusegem 2018).

Transition from motile spermatozoids in non-seed plants to sperm cell transport via pollen tubes in seed plants requires ROS-dependent signaling and recognition as well as very local and specific cell wall modifications. Pollen formation as well as pollen/stigma interactions involve ROS (Zhou and Dresselhaus 2023; Sankaranarayanan et al. 2020) and rapid tip growth in pollen is sustained by cell wall loosening via RBOH H and RBOH J in *A. thaliana* (Kaya et al. 2014; Mhamdi and van Breusegem 2018). While entering the female gametophyte, pollen tube rupture is initiated by hydroxyl radical formation triggered via the FERONIA (FER) LRR (Duan et al. 2014; Wolf et al. 2022). During double fertilization and concomitant cell deaths, the role of ROS dynamics is under investigation (Ali and Muday 2024; Zhou and Dresselhaus 2023).

Although the involvement of several RBOH isoforms in land plant development and reproduction points to an important role for the apoplastic redox balance, extracellular redox processes including the cell wall as a hydrated polymeric material with different components are largely unexplored (Cosgrove 2022). Regarding development, it is known that the pre-lignin pathway present in bryophytes is important for cuticle formation that is required for organ separation by boundary formation in the moss *P. patens* (Renault et al. 2017). In addition, as in animal cells, steady state redox potentials of the extracellular space may be dynamically regulated, affecting processes such as proliferation, differentiation and cell death (Banerjee et al., 2012) that are also occurring during land plant reproduction. In general, protein cysteinyl redox states slowly equilibrate with the GSH redox potential *E*_GSH_, a reaction that can be catalyzed via class I glutaredoxins (Deponte 2017). Apoplastic GSH levels in plants have been determined in the range of few to ten percent of total levels, likely resulting from GSH export (Ohkama-Ohtsu et al. 2007; Zechmann et al., 2014; Foyer and Noctor 2016). Extracellular GSSG is enzymatically degraded (Ohkama-Ohtsu et al. 2007; Noctor et al., 2012) and extracellular *E*_GSH_ is expected to be similar or less reducing relative to the value measured in the secretory pathway of − 241 mV (Ugalde et al. 2022). Secreted proteins and peptides (e.g. RALFs and other cysteine-rich peptides) would thus contain mostly disulfides or otherwise oxidized forms of cysteines. To date, no enzymatic apoplastic reduction systems for cysteines are known (Meyer et al. 2021). It is unclear how dynamic cysteine redox states in peptides or proteins can be in the extracellular space or if potential redox changes may play biological roles. Thus, how extracellular redox steady states can be dynamically and specifically sensed is an open question. A first potential redox-responsive receptor kinase has been described: This cys-rich repeat (CRR) receptor-like kinases RLK (CRK) HPCA1 is involved in long distance signaling in response to stress (Fichman et al. 2022; reviews Castro et al. 2021; van Breusegem and Mittler, 2023).

The extent of extracellular redox modulation in plants is unclear, especially regarding specialized ROS functions during the different modes of land plant reproduction. As the term ROS is generic (Sies, 2022), a future challenge will be to link ROS-dependent development and fertility to the respective steady-state levels of the distinct ROS types and mechanisms of their generation, removal and sensing. Here, staining procedures for ROS are often not sufficiently specific to discriminate ROS types and are unable resolve temporal and compartmentalized dynamics. Similarly, mechanisms affecting cysteine thiol steady state oxidation levels in the apoplast await identification. Thus, dissection of redox-related processes during reproduction requires further investigation of redox dynamics in apoplastic proteins and peptides, as well as identifying the gene networks and modules that determine local redox environments in and beyond the RBOH protein family.

## Participation of redox signal pathways and hypoxia in plant sexual reproduction

Accumulating evidence has shown that intracellular ROS and other oxidative signals exert crucial regulatory functions during angiosperm development, encompassing seed and bud dormancy, root and shoot growth, meristem organization and reproductive processes such as flowering, fertilization, and seed formation (Considine and Foyer 2021; Ali and Muday 2024). In *Marchantia*, the TCP transcription factor (TF) Mp*TCP1* regulates a comprehensive downstream redox network that includes numerous deregulated class III peroxidases, which are known to function in the apoplastic generation and degradation of H_2_O_2_. Mp*TCP1* mutants exhibit a disrupted ROS balance and severe growth and reproductive defects (Busch et al. 2019). Notably, comparative analyses revealed class III peroxidases as an outstanding family that extremely diversified in Arabidopsis and Marchantia, suggesting their involvement in adaptive ancestral traits (Beaulieu et al. 2025).

Given the potentially detrimental effects of ROS, precise spatial and temporal regulation of ROS production and signal transmission is essential for normal plant growth and the formation of sexual organs (Huang et al. 2019). ROS signals are often transduced into redox reactions, electron transfer reactions that can modify protein activities. In addition to the direct involvement of ROS in redox reactions, small oxidoreductases of the thioredoxin-superfamily, such as glutaredoxins (GRX) or thioredoxins (TRX), exhibit additional transmitter-like roles (Dietz 2008). The transfer of redox signals to protein thiols that function as regulatory switches participate in various processes, including plant reproduction. These posttranslational redox modifications (PTMs) can alter protein functions through changes in biochemical activity, conformational integrity or intracellular localization (Considine and Foyer 2021; Traverso et al. 2013).

Originally, plant GRX and TRX functions were linked to maintaining metabolic balance and regulating redox homeostasis under stress (Meyer et al. 2008, Wu et al., 2017). The significance of GRX and TRX redox systems in plant reproduction became apparent upon analyzing corresponding mutants. In Arabidopsis, plant fertility is decreased in mutants lacking both *NTRA* and *NTRB* genes, the two NADPH dependent thioredoxin reductases responsible for transferring electrons from NADPH to TRX. Introducing a glutathione deficiency in these mutants severely interfered with meristem maintenance, growth, and flower development, indicating the importance of the interplay between the antioxidant buffer glutathione and the TRX system in plant reproduction (Reichheld et al. 2007; Bashandy et al. 2010). The Arabidopsis GRX *ROXY1*, a member of the land plant-specific CC-type GRX class, participates in the control of flower organ and root development. Flowers from *roxy1* mutant plants develop fewer petals and later exhibit defects in petal morphology (Xing et al. 2005). Moreover, *ROXY1* together with its closest homolog *ROXY2* exert redundant functions in the formation of the male germline. Double *roxy1 roxy2* mutants fail to produce fertile pollen and are male sterile (Xing et al., 2008). Expression studies suggest that CC-type GRX family members such as *ROXY7*, *ROXY10* and *ROXY21* are involved in female germline formation (Gutsche et al. 2015). Interestingly, *MSCA1* and *MIL1*, two *ROXY1* homologs from maize and rice, also function in anther development, mediating the switch from mitosis to meiosis and thus pollen formation (Fig. 3) (Hong et al. 2012; Chaubal et al. 2003; Kellhier and Walbot, 2012). The resulting male sterility in loss-of-function mutants like *msca1* is of economic interest for hybrid seed production (Wan et al. 2019; Traverso et al. 2013).

As GRXs are known to participate in redox signaling, identifying their targets for posttranslational modifications is of great interest. In Arabidopsis, TGACG-binding (TGA) TFs have been identified as nuclear ROXY interaction partners (Li et al. 2009; Ndamukong et al. 2007). Common TGA TF and ROXY activities in flower development and reproduction are also evident in other angiosperm species, including rice, cassava, and maize (Yang et al. 2015, Gutsche et al. 2017, Hong et al. 2012, Ruan et al. 2022, Zander et al., 2012, Kobayashi et al. 2024). Notably, recent in vitro studies unraveled a PTM mechanism involving ROXY and TGA TF proteins, demonstrating an electron transfer from the ROXY homolog MSCA1 to the TGA target TF FEA4 from maize (Yang et al. 2021). The presence of specific cysteine residues in both proteins is crucial for this redox modification, which affects the DNA binding capacity of FEA4 and, consequently, its regulatory activity in flower development.

Heterologous expression experiments demonstrated that ROXY homologs from the liverwort Marchantia and rice can complement the flower phenotype of *roxy1* Arabidopsi*s* mutants (Gutsche et al. 2017; Hong et al. 2012). The capability of land-plant specific ROXY CC-type GRXs to modulate—likely by electron transfer—target protein activities has thus been conserved during the evolution of land plants and contributed to the formation of increasingly complex reproductive structures. Super-resolution microscopy studies detected that nuclear Arabidopsis ROXY1 proteins co-localize with different isoforms of RNA polymerase II at a resolution level of < 50 nm, which is abrogated under oxidizing conditions (Gutsche et al. 2017; Maß et al. 2020). This suggests that ROXY1 may sense nuclear redox states and influence transcriptional regulation through PTM of regulators, as well as by modulating transcription elongation and termination processes. The expansion of the land plant-specific CC-type GRX group during evolution, along with their conserved activity, indicates that functional diversification and their recruitment into novel reproductive processes were mediated by *cis*-regulatory changes (Gutsche et al. 2015). The diversification of GRX expression patterns expanded their repertoire of TF interactions and posttranslational modifications and thereby the regulation of downstream networks.

Recently, the roles of developmentally regulated hypoxic niches—areas with reduced cellular oxygen levels—emerged as another important cue for the control of plant development (Leon et al., 2021). Hypoxic niches have been identified in the shoot apical meristem (SAM) as well as reproductive meristems and are crucial for their maintenance (Zheng et al., 2017, Weits, 2019). Low oxygen levels in the SAM regulate the transition to flowering and are also important for male germline development (Kellhier and Walbot, 2012; Weits et al. 2021). Transient hypoxic conditions during early maize anther primordia formation are required for archesporial cell production, while these conditions are disappearing later in development (Dukowic-Schulze and van der Linde 2021). The loss of these hypoxic conditions leads to ectopic differentiation and male sterility, demonstrating the importance of spatio-temporally restricted hypoxic conditions in maize tassel development. Hypoxic conditions induce the activity of the maize CC-type GRX MSCA1, which regulates germinal cell initiation resulting male sterility, connecting hypoxia and redox signaling in plant reproduction. The establishment of novel tools, such as advanced H_2_O_2_ sensors for in vivo ROS studies promises to shed light on the dynamics and interplay of ROS and hypoxia in plant sexual reproduction (Pak et al. 2020; Nietzel et al. 2019).

## Phytohormones in the evolution of plant reproduction 

Phytohormones are key to almost any developmental pattern observed in land plants. Unsurprisingly sexual reproduction, from onset of flowering to gamete development up to the formation of the embryo is controlled by a phytohormone network in angiosperms as well. Contrarily to the functional data from angiosperms, the evolutionary transitions for these phytohormones and their functions particularly in reproductive development outside angiosperms is less-well understood.

Auxins and Gibberellins (GAs) appear to be players in germline specification and development of angiosperms (Cai et al. 2023). GA signals via a GID1-DELLA complex (Ueguchi-Tanaka et al. 2005; Willige et al. 2007). Mutant studies, particularly on GID1 paralogs as well as expression lines under ovule-specific and 35S promoters, and GA sensors, led to the picture that GAs may regulate germline expansion (Cai et al. 2023). Additionally, phytohormone profiles of male and female cones of *Pinus koraiensis* further suggest that gymnosperms, too, use GAs during their reproductive development (Li et al. 2023). In agreement, the GA receptor GID1 was predicted to have originated from a duplication event in the LCA of tracheophytes (Yoshida et al. 2018). The same study showed that recognition of bioactive GAs is reduced in lycophyte GID1 and appears to increase in the ancestor of ferns and seed plants (Yoshida et al. 2018). Interestingly, a recent study on DELLA proteins suggests that the interaction of DELLA with transcription factors is conserved across land plants, although TF interaction partners can vary per lineage and is hypothesized to affect the processes GA regulates (Briones-Moreno et al. 2023). Coherently, analysis of chromosome-scale genomes of 11 hornworts found a GID1 ortholog in six of the hornwort genomes and their phylogenetic distribution suggests a secondary loss in the other hornworts (Schafran et al. 2025). Yet, this GID1 ortholog does not retain the GA-binding residues known from other land plants, despite that the alternative residues are conserved across hornworts (Schafran et al. 2025; Yoshida et al. 2018). This agrees with the situation observed in the model moss and liverwort, which produce *ent*-kaurenoic acid (KA), a precursor to GA (Miyazaki et al. 2018; Sun et al. 2023). In *M. polymorpha,* derivatives from KA are bioactive and mutants in KA biogenesis led to delayed gametogenesis and aberrant gametangiophores (Sun et al. 2023). Likewise, KA-biosynthesis mutants in *P. patens* influence transition to chloro- and caulonemata, which are cells that can induce budding and produce gametophores (Miyazaki et al. 2018). The absence of GID1 in *M. polymorha* led Sun and colleagues (2023) to suggest that bioactive KA derivatives and bioactive GAs of angiosperms were independently recruited. With the recent data from hornworts, this assumption may have to be revisited.

In contrast to GA, auxin has a maximum at the apical cells of the ovule primordium of angiosperms (Ceccatto et al. 2013). Mutants in auxin biosynthesis (*YUC1*), export (*PIN1*) and regulation of auxin responses (miR160, miR390*, ARF3* and *17,* and tasiR-ARF) change the numbers of megaspore mother cells (MMC) per ovule and influence the second and/or third meiotic stage of the MMC (Su et al. 2017, 2020; Huang et al. 2022). Auxins are biosynthesized by many organisms other than plants, yet the biosynthesis genes known from *A. thaliana* are not fully conserved across streptophytes (Carrillo-Carrasco et al. 2023). The full biosynthesis pathway, however, was present in the LCA of land plants as was auxin transport and signaling (Carrillo-Carrasco et al. 2023). Indeed, manipulation of miR390 signaling in *P. patens* alters budding (Cho et al. 2012), but the canonical mechanism by which miR390 regulates ARF3 first emerged in the LCA of vascular plants (Xia et al. 2017). In *P. patens* the main auxin is indole-3-pyruvic acid (Landberg et al. 2021). Despite miR390 controlling bud-formation, the transition to the reproduction appears not to require auxin (Cho et al. 2012, Landberg et al. 2021). Yet, the tryptophan aminotransferase *PpTAR* mutants lead to increased number of antheridia, which is due to ectopic antheridia formation (Landberg et al. 2021). In *M. polymorpha* antheridiophores and antheridia have high levels of auxin according to the GUS distribution in *MpIAA:GUS* lines (Kato et al. 2015). This pattern was not observed in the female gametangiophores and gametangia. When auxin sensing was repressed, however, affects stalk length of gametangiophores (Kato et al. 2015). If the apical notch shows reduced levels of auxin, dormancy of gemmae was lost (Eklund et al. 2015). Interestingly, treatment with the auxins, indole-3-acetic acid (IAA) and 1-Naphtaleneacetic acid (NAA), on *C. richardii* immature gametophytes results in disorganization of the apical notch, albeit not exactly the phenotype observed for *M. polymorpha* (Woudenberg et al. 2024).

Data on Brassinosteroids (BRs) are less clear, but BRs appear to also contribute to the female germline of *A. thaliana* (Cai et al. 2023). The current hypothesis is that BRs define the female germline identity to one cell by restricting it in the cells next to the MMC (Cai et al. 2022). The phytohormone profiling on *Pinuskoraiensis* further supported a role of BRs in cone development (Li et al. 2023). BRs have been detected in bryophytes, lycophytes and ferns (Yokota et al. 2017). A homolog transcriptional regulator of BR signaling BRI1-EMS-SUPPRESSOR 1 (BES1) in *M. polymorpha*, *MpBES1* was investigated by Mecchia et al. (2021), knockdown and knockout plants of *MpBES1* produced neither gemmae cups nor antheridiophores after treatment with far-red light. Moreover, Burow et al. (2024) identified a brassinosteroid receptor-like kinase as a crucial contributor to male sex determination in undetermined gametophytes of *C. richardii* through analysis of antheridiogen-insensitive mutants*.* Antheridiogen is produced by the hermaphrodite and secreted, resulting in the induction of transition to male gametophytes in homosporous ferns (Döpp 1950). The study further showed that that BR biosynthesis pathway is induced in hermaphrodite vs. male gametophytes (Burow et al. 2024). Agreeingly, female gametophytes of *Dryopteris oreades* accumulated the brassinosteroid castasterone compared to gametophytes of asexual D*ryopteris affinis* ssp. *affinis* (Fernández et al. 2021). Other phytohormones, such as the auxin IAA, several cytokinins, GA_4_ and abscisic acid (ABA) are also enriched in the sexual vs. apogamous *Dryoteris* species (Fernández et al. 2021). A study on the latter showed that silencing of *CrSnRK2* has a reduced sensitivity to ABA (McAdam et al. 2016). In *C. richardii* antheridiogen-induced switch to male gametophytes can be blocked by treatment with ABA (Hickok 1983). In *CrSnRK2*silenced plants this effect is not seen, and male hermaphrodites develop in the presence of ABA. Moreover, ABA treatment of *SnRK2* mutant lines shows of *C. richardii* reduces ABA-dependent inhibition of spore germination (McAdam et al. 2016). Indeed, ABA also regulates spore germination in *P. patens* (Moody et al 2016; Vesty et al. 2016).

Mutants in jasmonic acid (JA) biosynthesis and signaling have strong effects on fertility (Huang et al. 2017). In maize aberrant JA biosynthesis and perception leads to the formation of female and female-like organs rather than male tassels (Yan et al. 2012). In *Arabidopsis*, JA biosynthesis and signaling mutants show disrupted anthesis and affect viability of pollen (Huang et al. 2017), JA mutants in rice are male sterile (Pak et al. 2020). Jasmonates are produced in all land plants and even some streptophyte algal lineages (Chini et al. 2023; Schmidt et al. 2024). Yet, the production of bioactive JA-Ile is restricted to tracheophytes, while the production of the jasmonate 12-oxo-phytodienoic acid (OPDA) and downstream derivatives has an embryophyte-wide distribution (Chini et al. 2023). Coherently, in the bryophyte *M. polymorpha* the jasmonate receptor CORONATINE-INSENSITIVE 1 (COI1) recognizes the derivative *dinor-*OPDA (*dn-*OPDA) instead of JA-Ile (Monte et al. 2018). OPDA biosynthesis mutants Δ*PpAOC1* and Δ*PpAOC2* show reduced fertility in the moss *P. patens* (Stumpe et al. 2010); pointing to a conserved role of jasmonates in fertility. Surprisingly, *Mpcoi1* had no effect on female or male fertility in *M. polymorpha* (Monte et al. 2018)*.* This suggests three possibilities: (i) jasmonate-control over fertility is lost in *M. polymorpha,* (ii) *M. polymorpha* uses a COI1-independent pathway for jasmonate-controlled fertility, or (iii) COI1-dependent signaling to regulate fertility has evolved after the split of bryophytes and tracheophytes. Indeed, a COI1-independent pathway for jasmonate signaling exists and appears to have been present in the LCA of Klebsormidiophyceae and the Phragmoplastophyta (Monte et al. 2020).

Consistent with jasmonates controlling male fertility, salicylic acid (SA) is involved in the formation of female reproductive organs. Transcriptomic and metabolomic analyses of male and female flowers, hints that SA-triggered programmed cell death leads to the degeneration of tapetum cells, followed by stamen abortion in dioecious plants, and female flower development in *Vernicia fordii *(Liu et al. 2019). In rice on the other hand, exogenous treatment of SA reduced programmed cell death of tapetum cells during heat stress, resulting in less accumulation of ROS and reduced stamen abortion (Feng et al. 2018). SA is present in all land plants in varying levels (Jia et al. 2023). Biosynthesis of SA has two main routes, of which synthesis via benzoic acid pathway may be the ancient one (Jia et al. 2023); yet only one fern *Azolla* has lost the first enzyme, isochorismate synthase, of the chorismate-derived route (de Vries et al. 2023). SA is bound and perceived via Non-expressor of pathogenesis related genes (NPR) (Wang et al. 2020; Jeon et al. 2024). In *M. polymorpha Mp*NPR act together with the TGAGC-motif binding transcription factor *Mp*TGA to regulate sexual reproduction (Gutsche et al. 2024). The study by Gutsche and colleagues (2024) showed that knockout mutants of Mp*TGA* fail to initiate and form male and female gametangiophores (Gutsche et al. 2024). Mp*npr* lines in this study were also affected, showing delayed reproductive organ development and a reduced number of male and female gametangiophores. Together, this shows that in both bryophytes and angiosperms SA is integrated into sexual reproduction. Yet, the mechanism with which may be different and so maybe the exact processes SA influences. Further research is needed to elucidate the conserved and distinct functional roles as SA exhibits in the diversity of the green lineage.

In summary, it is evident that phytohormones play crucial roles for regulating reproductive development across the green lineages. However, their roles may have been co-opted through evolutionary time not facilitating the same exact functions in different lineages.

## Conclusions

Here we reviewed the evolutionary adaptations that allow plants to successfully reproduce on land in a water-dependent as well as a water-independent manner. We highlighted well-known key innovations such as the development of pollen, loss of sperm motility and the evolution of seeds and carpels. These innovations were facilitated by co-evolution of molecular mechanisms that guide sexual reproduction, regulating, among others, gamete/gametophytic signaling, small RNA-mediated transcription and ROS-associated posttranslational and cellular responses. Here, knowledge generated by different fields of research starts to assemble into a comprehensive picture linking key aspects of land plant reproductive evolution. Successive molecular changes leading to novel tissues and organs require co-evolution of communication systems between tissues, sometimes being even highly specific, allowing discrimination among individuals of the same species. Taken together, these adaptations have re-shaped the water-dependent reproduction of the last common ancestor of land plants to a water-independent type of reproduction in the last common ancestor of seed plants. They were vital for plant fitness in diverse terrestrial habitats and significantly contributed to the evolutionary success of seed plants.

To understand how such water-independence was achieved, genetic networks shaping ovule and carpel development were investigated in *A. thaliana* extensively, yet the genetic mechanisms behind ovule, seed, and carpel origin remain, to the largest part speculative. We know even less about when these networks have originated and how they have been modified in different lineages and across evolutionary time. Here, we outline that the emergence of ovules and carpels with their accompanying gene regulatory networks likely evolved through the integration of pre-existing transcriptional modules, with whole genome duplications playing a critical role in diversifying and shaping these networks to what we see today in extant flowering plants. Co-evolution of modules as well as co-option of existing molecular mechanisms is likely key to this as can be seen from on various examples in this review. However, only few genes and their interactions have been functionally tested in the new non-angiosperm genetic model organisms. This will be one of the key next steps to understand the evolutionary history of reproductive networks and to identify the origins of key denominators of water-independent reproduction.
